# Late-Stage Copper-Catalyzed Radiofluorination of an Arylboronic Ester Derivative of Atorvastatin

**DOI:** 10.3390/molecules24234210

**Published:** 2019-11-20

**Authors:** Gonçalo S. Clemente, Tryfon Zarganes-Tzitzikas, Alexander Dömling, Philip H. Elsinga

**Affiliations:** 1 Department of Nuclear Medicine and Molecular Imaging, University Medical Center Groningen, University of Groningen, 9713 GZ Groningen, The Netherlands; 2 Department of Drug Design, Groningen Research Institute of Pharmacy, University of Groningen, 9713 AV Groningen, The Netherlands

**Keywords:** fluorine-18, radiochemistry, late-stage radiofluorination, drug development, copper-catalyzed, boronic pinacol ester

## Abstract

There is an unmet need for late-stage ^18^F-fluorination strategies to label molecules with a wide range of relevant functionalities to medicinal chemistry, in particular (hetero)arenes, aiming to obtain unique in vivo information on the pharmacokinetics/pharmacodynamics (PK/PD) using positron emission tomography (PET). In the last few years, Cu-mediated oxidative radiofluorination of arylboronic esters/acids arose and has been successful in small molecules containing relatively simple (hetero)aromatic groups. However, this technique is sparsely used in the radiosynthesis of clinically significant molecules containing more complex backbones with several aromatic motifs. In this work, we add a new entry to this very limited database by presenting our recent results on the ^18^F-fluorination of an arylboronic ester derivative of atorvastatin. The moderate average conversion of [^18^F]F^−^ (12%), in line with what has been reported for similarly complex molecules, stressed an overview through the literature to understand the radiolabeling variables and limitations preventing consistently higher yields. Nevertheless, the current disparity of procedures reported still hampers a consensual and conclusive output.

## 1. Introduction

Being already a clinically established molecular imaging modality, positron emission tomography (PET) increasingly broadened its application field by also becoming an essential partner of the pharmaceutical industry [1,2]. Its unique combination of spatial resolution, quantification, and detection sensitivity provides essential in vivo information at an early stage by directly measuring tissue uptake concentrations of the radiolabeled molecules of interest. Ideally, the radionuclide should be added to the desired molecular structure causing as little disturbance as possible, especially in the vicinity of the active site(s), and at the latest possible stage in the process to avoid radiation loss and exposure. Historically, radiochemistry found an unparalleled ally in nucleophilic substitution reactions with [^18^F]F^−^ [3,4]. However, this became more challenging when the focus fell on the labeling of (hetero)arenes that are not easily reactive to aromatic nucleophilic substitutions. The ubiquitous role of heteroaromatic pharmacophores in drug development and medicinal chemistry stressed out the need for improved radiofluorination techniques to overcome the typically far-from-ideal electrophilic fluorination with carrier-added [^18^F]F_2_. Recently, several methods have been published aiming for a practical, transversal, and straightforward ^18^F-fluorination of electron-rich, -poor, and –neutral (hetero)arenes [5,6,7,8,9,10,11,12,13,14,15]. One of these strategies, the late-stage copper-mediated oxidative ^18^F-fluorination of arylboronic ester and acid derivatives, has received great attention from radiochemistry research groups but is still not routinely applied in the production of clinical PET radiopharmaceuticals. Numerous basic-research proposals for improving this Cu-catalyzed reaction have been successfully reported and conceptualized with simple heteroaromatic groups [15,16,17,18,19,20,21,22,23,24,25,26,27,28,29,30,31,32,33], but advanced applications to more complex molecules with potential clinical value are sparse and generally reveal very fluctuating ^18^F-fluorination efficiencies [33,34,35,36,37,38]. Following previous work from our group [39], where we applied this Cu-mediated strategy to several structurally different drug-like molecules and investigated the influence of a range of temperature, solvents, catalyst, and precursor amounts, we aimed to go up in terms of complexity, applicability, and relevance. As a proof-of-concept, we synthesized an arylboronic ester derivative of atorvastatin (**6**), the highest-selling drug of all time and one of the most clinically prescribed. The presence of three phenyl groups and an electron-rich pyrrole core, together with a flexible hydrophobic side-chain, entails an increasingly challenging ^18^F-fluorination test to this Cu-catalyzed strategy when compared to our previous simple drug-like molecules or even to the majority of the molecules reported in the literature. Thus, to highlight the potentialities and drawbacks of this radiolabeling strategy, herein we present and discuss one of the most complex labeling precursor scaffolds that have been submitted to Cu-catalyzed radiofluorination. With this, we add a new and significant entry to the still very structure-limited database of bioactive molecules that have been radiolabeled via this strategy. Moreover, the existence of a radiolabeled atorvastatin analog has the potential to become a widespread research tool to aid in the understanding of the recently reported pleiotropic and off-target mechanisms of statins [40,41], enabling the study of cellular and subcellular interactions through high sensitive nuclear analytical and imaging techniques. The findings using [^18^F]atorvastatin (**8**) may then be inferred to the native molecule increasing the knowledge related to its pharmacokinetics/pharmacodynamics, which represents a practical example of the synergy that can exist between PET imaging and the pharmaceutical industry. As atorvastatin is a widely characterized and registered drug, any envisaged clinical assays with this radiotracer are also facilitated by the fact that its toxicological profile is already well described.

## 2. Results

**Scheme 1:**
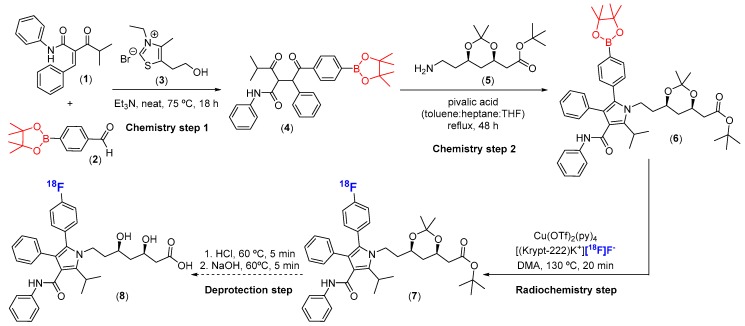
Synthesis route of Bpin precursor (**6**) and radiolabeling approach used in this work.

The introduction of a labile boronic pinacol ester (Bpin) in the position to be radiofluorinated, facilitates the intermediate transmetalation with [Cu(OTf)_2_(py)_4_] and further coordination to [^18^F]F^-^, to yield, after oxidation and reductive elimination, the desired [^18^F]fluorobenzene derivative (Scheme 1). With this procedure, a radioactive analog of the atorvastatin intermediate (**7**) was synthesized since the original structure is preserved with the native fluorine being solely substituted by its β^+^-emitting radioisotope (conserving physicochemical and biological properties).

For the ^18^F-fluorination of the Bpin labeling precursor (**6**), the aqueous [^18^F]F^−^ produced in a biomedical cyclotron was quantitatively trapped (>95%) in an anion-exchange cartridge. The presence of an excess of basic salts and phase-transfer agents, typically used to efficiently recover the trapped [^18^F]F^−^ and enhance its reactivity, are known to be detrimental to the Cu catalyst stability, disturbing the essential oxidation/reduction cycle for the radiolabeling. Thus, to not significantly affect [^18^F]F^−^ elution, a previously optimized [39] balanced compromise was achieved using 3.15 mg of kryptofix 2.2.2 (Krypt-2.2.2), 50 µg of K_2_CO_3_, and 0.5 mg of K_2_C_2_O_4_ in 1 mL 80% CH_3_CN (elution efficiency: 80.3% ± 2.5%, *n* = 7, when performed dropwise). The recovered [(Krypt-2.2.2)K^+^][^18^F]F^−^ solution was then azeotropically dried at 105 °C under gentle magnetic stirring and a light stream of argon (directly over the solution and not in the solution), without ever letting the mixture to completely dry. The softness of this drying step seems to be important to minimize the often significant losses of activity by evaporation and adsorption of the [^18^F]F^−^ to the borosilicate glass reaction vial walls (Table S1). After increasing the temperature to 130 °C and adding an optimized [39] solution of 60 µmol of Bpin labeling precursor (**6**) and 20 µmol of [Cu(OTf)_2_(py)_4_] in 0.8 mL dimethylacetamide (DMA), the reaction mixture was left to react under vigorous stirring for 20 min, as increasing the reaction up to 60 min only improved the final [^18^F]F^−^ conversion yield by approximately 3%. At the very beginning and after 10 min of the reaction, the sealed vial was purged with 5 mL of dried atmospheric air (passed through a P_2_O_5_ cartridge) to facilitate the re-oxidation of the copper complex, as the Cu(III) species seem to be responsible for the nucleophilic aromatic substitution [33,42]. However, this procedure does not appear to be relevant for the success of the reaction as the absence of it led to identical radiolabeling results.

Although achievable, the approach used only yielded an inconsistent radiofluorination of the labeling precursor (12% ± 11% determined by multiplying the radio-TLC conversion of [^18^F]F^−^ with radio-HPLC purity, *n* = 7). The absence of products of degradation and radiochemical impurities from the chromatographic spectra (Figure S3), associated with the still visible signal of the intact Bpin labeling precursor (**6**), suggests that the ^18^F-fluorination might have been hampered by a reduction of the Cu catalyst reactivity. It is known from the literature that the atorvastatin side chain [43], the presence of the two non-functionalized mono-substituted benzene rings and a pyrrole core [44], and the basic salts in solution [16] can all influence copper oxidation states, which might explain the limited [^18^F]F^−^ conversion. Nevertheless, the radiofluorination yields obtained in this work are in line with what has been reported for complex heteroaromatic molecules, especially if containing several phenyl groups in its structure [33,35,38], and should still be sufficient to proceed for the development of [^18^F]atorvastatin (**8**) preclinical screening assays after a fast and nearly quantitative deprotection of the side chain [45] (Figure S4).

## 3. Discussion

The Cu-mediated oxidative ^18^F-fluorination strategy improved the radiochemistry field by supplying a practical solution for the labeling of (hetero)arenes. The proof-of-concept radiofluorination of arylboronic esters and acid derivatives, without the presence of extensive heteroaromatic functional groups, has already been proven successful. But the translation to larger scales and more complex biologically active molecules aimed for PET application/evaluation is generally associated with low to moderate ^18^F-fluorination yields and reproducibility. The radiofluorination herein presented with an arylboronic ester derivative of atorvastatin (**6**) proved to be in line with these findings and led us to an overall review through the literature to understand the radiolabeling variables and limitations preventing consistently higher yields. This late-stage Cu-mediated radiofluorination strategy has already shown to be very dependent of the type and complexity of the labeling precursor used, and very sensitive to all the processes associated with the method–from the additives used to enable [^18^F]F^−^ elution, passing through the azeotropic drying harshness, the anhydrous environment level, and reaction solvents used, to the temperature, reagent amounts and Cu catalyst type. The base and phase transfer catalyst amounts used for the radiolabeling of the atorvastatin intermediate have been previously optimized in our latest work [39]. Higher amounts invariably ended up in no detecTable ^18^F-fluorination of the Bpin atorvastatin precursor (possibly due to the formation of copper adducts [22]), and lower amounts resulted in poorer elution efficiencies without improving the final [^18^F]F^−^ conversion yield to the radiofluorinated product. The softness of the drying step can also be essential for the procedure to not fall in one of the drawbacks of this radiolabeling methodology—the significant reduction of [^18^F]F^−^ availability for the reaction due to the extensive escape of activity and adsorption to the borosilicate glass reaction vial walls. In our work, we reached the best results by preventing the [(Krypt-2.2.2)K^+^][^18^F]F^−^ solution from tumultuous boiling and harsh agitation, as this avoids splashing of the complex to upper regions of the reaction vial that will not be in contact with the subsequent Bpin labeling precursor/Cu-catalyst solution. Additionally, it is also beneficial to not let the [(Krypt-2.2.2)K^+^][^18^F]F^−^ solution completely evaporate (3 azeotropic drying cycles with 0.5 mL anhydrous acetonitrile, each one starting after the previous volume has almost vanished, followed by the dilution with 100 µl of anhydrous DMA immediately after the last cycle has nearly evaporated completely and the further addition of the remaining solvent with the precursor (**6**) and [Cu(OTf)_2_(py)_4_]). To circumvent the downsides of azeotropic evaporation, especially when automated where manipulation and close control of the conditions are challenging, a few solid-phase extraction (SPE) drying procedures have been rising in the literature [21,27,28,30], some even able to avoid the use of bases [29]. However, being very recent, they still lack a proper multicentre evaluation and assessment into more than just simple (hetero)arenes, as some authors claim not being able to reproduce them [38] and when attempted by us for the ^18^F-fluorination of the arylboronic ester derivative of atorvastatin (**6**), invariably led to no detectable [^18^F]F^−^ conversion (despite shown to be successful when tested first in some of the same simple aryl boronic acid esters used in our previous work [39]).

Currently, late-stage Cu-mediated ^18^F-fluorination of precursors containing multi (hetero)arenes in their structure is still very dependent on a range of variables and on the existing expertise in the radiochemistry lab performing it. Therefore, a case-by-case optimization still seems to be necessary, being extremely difficult to reach a standardized procedure for every labeling precursor, which might explain the reason why the exact same methodology has been very rarely repeated in the literature. An analysis through the Cu-mediated works published, and hereby referenced [15,16,17,18,19,20,21,22,23,24,25,26,27,28,29,30,31,32,33,34,35,36,37,38,39], shows that [Cu(OTf)_2_(py)_4_] is still by far the most common catalyst used (against other options such as Cu(OTf)_2_, Cu(OTf)_2_(associated with diverse pyridine derivatives), or Cu(CF_3_SO_3_)_2_), and the typical amounts for all of them are between 5 to 30 µmol while the Bpin labeling precursor may vary from 4 to 60 µmol. The reaction temperatures are usually kept around 120 °C ± 10 °C while anhydrous DMA and dimethylformamide (DMF) are the solvents almost exclusively reported, with the first one having the propensity for better conversion efficacies [39] which can arguably be due to its higher boiling point and resistance to bases. Numerous base additives (e.g., potassium oxalate/trifluoromethanesulfonate, dimethylaminopyridine, tetraethylammonium bicarbonate/bromide, tetrabutylammonium fluoride, and trichlorophenylethenesulfonate) have been used for [^18^F]F^−^ elution, with potassium carbonate being preferentially chosen. Carrier-added ([^19^F]KF) radiolabeling reactions to simulate conventional fluorinations showed no improvement in the conversion yields [24]. The reaction times reported are typically between 20 to 30 min, and an experiment prolonging the reaction with the arylboronic ester derivative of atorvastatin (**6**) until 60 min did not result in a significant increase in [^18^F]F^−^ conversion.

In summary, from the analysis of the literature, a general association can be established between a higher concentration of reactants (and typically a 5 to 12 eq. excess of simple arylBpin precursor over [Cu(OTf)_2_(py)_4_]), and minimizing the reaction volume and the molar ratio of the added base, with ^18^F-fluorination efficiency. Nevertheless, the direct conversion of these conditions is not always practically (and economically) achievable for complex and clinically relevant (hetero)arene precursors, since this may result in the use of several dozens of mg of valuable precursor (as it happens with the current arylboronic ester derivative of atorvastatin (**6**)) instead of just a few mg of the simple arenes. This is also expected to have a negative impact on the final molar activity (GBq.mmol^−1^) of the radiotracer. Furthermore, the extensive use of a Cu-catalyst might bring additional issues, in terms of by-product formation and the need for further refined purification techniques.

## 4. Materials and Methods

### 4.1. General Procedure for the Synthesis of the Arylboronic Pinacol Ester (Bpin) Labeling Precursor (**6**)

Solvents and reagents, including the atorvastatin intermediate standard and atorvastatin reference (CAS 125971 95-1 and CAS 344423-98-9 from TCI Chemicals, Zwijndrecht, Belgium), were available from commercial suppliers and used without any further purification.

A mixture of 2-benzylidene-4-methyl-3-oxo-*N*-phenylpentanamide (**1**, 5 g, 17 mmol, 1.00 equiv.), 3-ethyl-5-(2-hydroxyethyl)-4-methyl-3-thiazolium bromide (**3**, 1.7 g, 6.8 mmol, 0.40 equiv.), triethylamine (5 mL, 36 mmol, 2.12 equiv.), and 4-formylphenylboronic acid pinacol ester (**2**, 4.9 g, 21 mmol, 1.20 equiv.) was heated at 75 °C under argon atmosphere with vigorous stirring for 16 h. The reaction was monitored by thin-layer chromatography (TLC) until the consumption of the *N*-phenylpentanamide (**1**). Isopropyl alcohol (25 mL) was added, and the reaction mixture was maintained at 25 °C for 4 h under stirring. The remaining solid was vacuum filtered and washed with 25 mL of water followed by 20 mL of isopropyl alcohol. The product was dried under high vacuum for 4 h, affording 4-methyl-3-oxo-2-(2-oxo-1-phenyl-2-(4-(4,4,5,5-tetramethyl-1,3,2- dioxaborolan-2-yl)phenyl)ethyl)-*N*-phenylpentanamide (**4**) as a yellowish crystalline solid in approximately 14% yield (1.8 g, 2.4 mmol).

Pivalic acid (0.5 g, 4.9 mmol, 3.77 equiv.) was added, under nitrogen atmosphere, to a solution of the previously synthesized phenylpentanamide derivative (**4**, 1 g, 1.3 mmol, 1.00 equiv.) and tert-butyl 2-((4R,6R)-6-(2-aminoethyl)-2,2-dimethyl-1,3-dioxan-4-yl)acetate (**5**, 1 g, 3.7 mmol, 2.85 equiv.) in toluene:heptane:tetrahydrofuran (1:4:1 *v*/*v*) (20 mL). The reaction mixture was refluxed for 24 h with azeotropic removal of water, monitored by TLC, cooled to room temperature, and extracted with ethyl acetate (3 × 50 mL). The organic phase was washed with saturated aqueous sodium chloride solution (50 mL). The solvent was removed under vacuum, the desired Bpin labeling precursor (**6**) was obtained as a pale yellow solid in approximately 60% yield (0.6 g, 0.8 mmol) after purification by column chromatography (petroleum ether:ethyl acetate).

### 4.2. Characterization Data

*tert-butyl 2-((4R,6R)-6-(2-(2-isopropyl-4-phenyl-3-(phenylcarbamoyl)-5-(4-(4,4,5,5-tetramethyl-1,3,2-dioxa- borolan-2-yl)phenyl)-1H-pyrrol-1-yl)ethyl)-2,2-dimethyl-1,3-dioxan-4-yl)acetate (Bpin labeling precursor **6**):*

^1^H NMR (500 MHz, CDCl_3_) δ 7.72 (d, *J* = 7.9 Hz, 2 H), 7.20–7.15 (m, 9 H), 7.06 (d, *J* = 7.9 Hz, 2 H), 6.97 (t, *J* = 7.4 Hz, 1 H), 6.88 (s, 1 H), 4.17–4.07 (m, 2 H), 3.91–3.82 (m, 1 H), 3.67–3.57 (m, 2 H), 2.35 (dd, *J* = 15.2, 7.3 Hz, 1 H), 2.22 (dd, *J* = 15.2, 5.8 Hz, 1 H), 1.72–1.58 (m, 2 H), 1.53 (dd, *J* = 7.1, 3.9 Hz, 6 H), 1.43 (s, 9 H), 1.34 (d, *J* = 2.6 Hz, 9 H), and 1.23 (s, 9 H).

^13^C NMR (126 MHz, CDCl_3_) δ 184.5, 170.3, 164.9, 141.8, 138.4, 135.1, 134.7, 134.6, 130.6, 130.6, 129.9, 128.6, 128.3, 126.5, 123.5, 121.7, 119.6, 115.4, 98.7, 83.9, 80.7, 66.4, 65.9, 42.5, 40.9, 38.5, 38.0, 35.9, 29.9, 28.1, 27.0, 26.0, 24.9, 24.5, 21.7, 21.6, and 19.7.

HRMS-ESI: *m*/*z* calcd. for C_46_H_60_BN_2_O_7_ [M + H]^+^ 763.452, found 763.379.

### 4.3. General Procedure for the Cu-mediated Radiosynthesis

All procedures involving the handling of radioactive substances were carried out in a radiochemistry laboratory with the required conditions of radiological protection and safety.

The Cu-mediated radiolabeling procedure followed our previously optimized method using several structurally different drug-like molecules functionalized with a Bpin leaving group [39]. The aqueous [^18^F]F^−^ used in this work was produced by the ^18^O(p,n)^18^F nuclear reaction in an IBA (Ottignies-Louvain-la-Neuve, Belgium) Cyclone 18/9 cyclotron and then loaded (approx. 1.5 GBq) into a polystyrene-divinylbenzene in a HCO_3_^−^ anion exchange cartridge (Chromafix 45-PS-HCO_3_^−^) without the need of any preconditioning. The cartridge was then washed out to a 5 mL borosilicate glass Wheaton reaction V-vial (containing a stirring bar) with 1 mL of an 80% acetonitrile solution of 3.15 mg Krypt-2.2.2, 0.05 mg K_2_CO_3_, and 0.5 mg K_2_C_2_O_4_. This solution was submitted to azeotropic drying with subsequent additions of anhydrous acetonitrile at 105 °C to originate moistureless [(Krypt-2.2.2)K^+^][^18^F]F^−^. Then, 0.8 mL of DMA with the boronic pinacol ester derivative labeling precursor (**6**, 60 µmol) was added to this same vial with the previously dissolved [Cu(OTf)_2_(py)_4_] catalyst (20 µmol, 0.33 equiv.). This reaction mixture was left under vigorous stirring at 130 °C for 20 min to afford **7** after a total synthesis time of under 60 min (Figure S3). The conversion to the ^18^F-product was assessed by radio-TLC (TLC-SG developed in hexane:ethyl acetate (1:1 *v*/*v*), Rf([^18^F]F^−^) = 0.0–0.2 and Rf(**7**) = 0.8–1.0) and radio—High performance liquid chromatography (HPLC) (SymmetryPrep^TM^ C18 7 µm 7.8 × 300 mm; A: sodium acetate 0.05 M pH 4.7, B: acetonitrile; 0–4 min.: 90% A, 4–15 min.: 90% A to 20% A, 15–25 min.: 20% A to 5% A, 25–33 min.: 5% A 33–34 min.: 5% A to 90% A, 34–35 min.: 90% A; flow: 6 mL.min^−1^.; Rf(**8**) ≈ 16 min Rf(**7**) ≈ 23 min). As a proof-of-concept, **7** was converted to [^18^F]atorvastatin (**8**) by a fast (extra 10 min of synthesis time) and nearly quantitative deprotection of the side chain [45] with HCl followed by NaOH. The final product (**8**) was then isolated (approx. 25 MBq) by HPLC (Figure S4).

## 5. Conclusions

Despite being potentially attainable with the Cu-mediated ^18^F-fluorination strategy, our goal for an enhanced automatable approach to achieve [^18^F]atorvastatin (**8**) in a larger production scale with practical and sufficient yields will continue, as the ultimate purpose is to proceed for the development of preclinical screening assays and further clinical evaluation in humans. A deeper understanding of the crucial conditions to optimize the yields obtained with the Cu-catalysed radiofluorination was attempted but due to the disparity of data, procedures, and labeling precursors reported in the literature, it is hardly possible to reach to a consensual and accurate conclusion. From a review of the literature, it seems undeniable that the nature of the (hetero)arene labeling precursor plays a major role in the efficiency of ^18^F-fluorination. The wise approach still seems to be to perform an individual “one variable at a time” optimization for each scaffold to be radiolabeled, despite the fact that this might ignore the influence of multifactorial interactions [46]. Thus, the search for more robust late-stage radiofluorination procedures compatible with suitable heteroaromatic pharmacophores remains a very stimulating topic that, ultimately, can lead not only to refined radiopharmaceutical drug discovery but also to aid the pharmaceutical industry to evaluate pharmacokinetics/dynamics and better understand certain mechanisms of action.

## Supplementary Materials

The following are available online at https://www.mdpi.com/1420-3049/24/23/4210/s1, Figure S1: ^1^H NMR characterization of the Bpin labeling precursor, Figure S2: ^13^C NMR characterization of the Bpin labeling precursor, Figure S3: Chromatographic profile of the compounds used and synthesized, Figure S4: Chromatographic profile of [^18^F]atorvastatin (**8**), Table S1: Influence of azeotropic drying procedure in [^18^F]F^−^ availability to the radiolabeling reaction.

## References

[1] Elsinga P.H., van Waarde A., Paans A.M.J., Dierckx R.A.J.O. (2012). Trends on the Role of PET in Drug Development.

[2] Fernandes E., Barbosa Z., Clemente G., Alves F., Abrunhosa A.J. (2012). Positron emitting tracers in pre-clinical drug development. Curr. Radiopharm..

[3] Miller P.W., Long N.J., Vilar R., Gee A.D. (2008). Synthesis of ^11^C, ^18^F, ^15^O, and ^13^N radiolabels for positron emission tomography. Angew. Chem. Int. Ed..

[4] Jacobson O., Kiesewetter D.O., Chen X. (2015). Fluorine-18 Radiochemistry, Labeling Strategies and Synthetic Routes. Bioconjugate Chem..

[5] Teare H., Robins E.G., Kirjavainen A., Forsback S., Sandford G., Solin O., Luthra S.K., Gouverneur V. (2010). Radiosynthesis and Evaluation of [^18^F]Selectfluor bis(triflate). Angew. Chem. Int. Ed..

[6] Pretze M., Große-Gehling P., Mamat C. (2011). Cross-Coupling Reactions as Valuable Tool for the Preparation of PET Radiotracers. Molecules.

[7] Lee E., Kamlet A.S., Powers D.C., Neumann C.N., Boursalian G.B., Furuya T., Choi D.C., Hooker J.M., Ritter T. (2011). A Fluoride-Derived Electrophilic Late-Stage Fluorination Reagent for PET Imaging. Science.

[8] Ichiishi N., Brooks A.F., Topczewski J.J., Rodnick M.E., Sanford M.S., Scott P.J.H. (2014). Copper-Catalyzed [^18^F]Fluorination of (Mesityl)(aryl)iodonium Salts. Org. Lett..

[9] Neumann C.N., Hooker J.M., Ritter T. (2016). Concerted nucleophilic aromatic substitution with ^19^F− and ^18^F−. Nature.

[10] Makaravage K.J., Brooks A.F., Mossine A.V., Sanford M.S., Scott P.J.H. (2016). Copper-Mediated Radiofluorination of Arylstannanes with [^18^F]KF. Org. Lett..

[11] Beyzavi M.H., Mandal D., Strebl M.G., Neumann C.N., D.’Amato E.M., Chen J., Hooker J.M., Ritter T. (2017). ^18^F-Deoxyfluorination of Phenols via Ru π-Complexes. Acs Cent. Sci..

[12] Tredwell M., Preshlock S.M., Taylor N.J., Gruber S., Huiban M., Passchier J., Mercier J., Génicot C., Gouverneur V. (2014). A General Copper-Mediated Nucleophilic ^18^F Fluorination of Arenes. Angew. Chem. Int. Ed..

[13] Coenen H.H., Ermert J. (2018). ^18^F-labelling innovations and their potential for clinical application. Clin. Transl. Imaging.

[14] Deng X., Rong J., Wang L., Vasdev N., Zhang L., Josephson L., Liang S.H. (2019). Chemistry for Positron Emission Tomography: Recent Advances in ^11^C-, ^18^F-, ^13^N-, and ^15^O-Labeling Reactions. Angew. Chem. Int. Ed..

[15] Preshlock S., Tredwell M., Gouverneur V. (2016). ^18^F-Labeling of Arenes and Heteroarenes for Applications in Positron Emission Tomography. Chem. Rev..

[16] Zlatopolskiy B.D., Zischler J., Krapf P., Zarrad F., Urusova E.A., Kordys E., Endepols H., Neumaier B. (2015). Copper-Mediated Aromatic Radiofluorination Revisited: Efficient Production of PET Tracers on a Preparative Scale. Chem. A Eur. J..

[17] Mossine A.V., Brooks A.F., Makaravage K.J., Miller J.M., Ichiishi N., Sanford M.S., Scott P.J.H. (2015). Synthesis of [^18^F]Arenes via the Copper-Mediated [^18^F]Fluorination of Boronic Acids. Org. Lett..

[18] Preshlock S., Calderwood S., Verhoog S., Tredwell M., Huiban M., Hienzsch A., Gruber S., Wilson T.C., Taylor N.J., Cailly T. (2016). Enhanced copper-mediated ^18^F-fluorination of aryl boronic esters provides eight radiotracers for PET applications. Chem. Commun..

[19] Schäfer D., Weiß P., Ermert J., Castillo Meleán J., Zarrad F., Neumaier B. (2016). Preparation of No-Carrier-Added 6-[^18^F]Fluoro-l-tryptophan via Cu-Mediated Radiofluorination. Eur. J. Org. Chem..

[20] Giglio B.C., Fei H., Wang M., Wang H., He L., Feng H., Wu Z., Lu H., Li Z. (2017). Synthesis of 5-[(18)F]Fluoro-α-methyl Tryptophan: New Trp Based PET Agents. Theranostics.

[21] Zischler J., Kolks N., Modemann D., Neumaier B., Zlatopolskiy B.D. (2017). Alcohol-Enhanced Cu-Mediated Radiofluorination. Chem. A Eur. J..

[22] Mossine A.V., Brooks A.F., Ichiishi N., Makaravage K.J., Sanford M.S., Scott P.J.H. (2017). Development of Customized [^18^F]Fluoride Elution Techniques for the Enhancement of Copper-Mediated Late-Stage Radiofluorination. Sci. Rep..

[23] Blevins D.W., Kabalka G.W., Osborne D.R., Akula M.R. (2018). Effect of Added Cu(OTf)2 on the Cu(OTf)2(Py)4-Mediated Radiofluorination of Benzoyl and Phthaloylglycinates. Nat. Sci..

[24] Zhou D., Chu W., Katzenellenbogen J. (2018). Exploration of alcohol-enhanced Cu-mediated radiofluorination towards practical labeling. J. Nucl. Med..

[25] Mossine A.V., Brooks A.F., Bernard-Gauthier V., Bailey J.J., Ichiishi N., Schirrmacher R., Sanford M.S., Scott P.J.H. (2018). Automated synthesis of PET radiotracers by copper-mediated ^18^F-fluorination of organoborons: Importance of the order of addition and competing protodeborylation. J. Label. Compd. Radiopharm..

[26] Lahdenpohja S., Keller T., Rajander J., Kirjavainen A.K. (2019). Radiosynthesis of the norepinephrine transporter tracer [^18^F]NS12137 via copper-mediated ^18^F-labelling. J. Label. Compd. Radiopharm..

[27] Antuganov D., Zykov M., Timofeev V., Timofeeva K., Antuganova Y., Orlovskaya V., Fedorova O., Krasikova R. (2019). Copper-Mediated Radiofluorination of Aryl Pinacolboronate Esters: A Straightforward Protocol by Using Pyridinium Sulfonates. Eur. J. Org. Chem..

[28] Zhang B., Fraser B.H., Klenner M.A., Chen Z., Liang S.H., Massi M., Robinson A.J., Pascali G. (2019). [^18^F]Ethenesulfonyl Fluoride as a Practical Radiofluoride Relay Reagent. Chem. A Eur. J..

[29] Zhang X., Basuli F., Swenson R.E. (2019). An azeotropic drying-free approach for copper-mediated radiofluorination without addition of base. J. Label. Compd. Radiopharm..

[30] Zlatopolskiy B.D., Zischler J., Krapf P., Richarz R., Lauchner K., Neumaier B. (2019). Minimalist approach meets green chemistry: Synthesis of ^18^F- labeled (hetero)aromatics in pure ethanol. J. Label. Compd. Radiopharm..

[31] Mossine A.V., Tanzey S.S., Brooks A.F., Makaravage K.J., Ichiishi N., Miller J.M., Henderson B.D., Skaddan M.B., Sanford M.S., Scott P.J.H. (2019). One-pot synthesis of high molar activity 6-[^18^F]fluoro-l-DOPA by Cu-mediated fluorination of a BPin precursor. Org. Biomol. Chem..

[32] Lahdenpohja S.O., Rajala N.A., Rajander J., Kirjavainen A.K. (2019). Fast and efficient copper-mediated ^18^F-fluorination of arylstannanes, aryl boronic acids, and aryl boronic esters without azeotropic drying. Ejnmmi Radiopharm. Chem..

[33] Taylor N.J., Emer E., Preshlock S., Schedler M., Tredwell M., Verhoog S., Mercier J., Genicot C., Gouverneur V. (2017). Derisking the Cu-Mediated ^18^F-Fluorination of Heterocyclic Positron Emission Tomography Radioligands. J. Am. Chem. Soc..

[34] Drerup C., Ermert J., Coenen H.H. (2016). Synthesis of a Potent Aminopyridine-Based nNOS-Inhibitor by Two Recent No-Carrier-Added ^18^F-Labelling Methods. Molecules.

[35] Lien V.T., Klaveness J., Olberg D.E. (2018). One-step synthesis of [^18^F]cabozantinib for use in positron emission tomography imaging of c-Met. J. Label. Compd. Radiopharm..

[36] Bernard-Gauthier V., Mossine A.V., Mahringer A., Aliaga A., Bailey J.J., Shao X., Stauff J., Arteaga J., Sherman P., Grand′Maison M. (2018). Identification of [^18^F]TRACK, a Fluorine-18-Labeled Tropomyosin Receptor Kinase (Trk) Inhibitor for PET Imaging. J. Med. Chem..

[37] Wilson T., Xavier M.-A., Knight J., Verhoog S., Torres J.B., Mosley M., Hopkins S., Wallington S., Allen D., Kersemans V. (2018). PET imaging of PARP expression using [^18^F]olaparib. J. Nucl. Med..

[38] Guibbal F., Meneyrol V., Ait-Arsa I., Diotel N., Patché J., Veeren B., Bénard S., Gimié F., Yong-Sang J., Khantalin I. (2019). Synthesis and Automated Labeling of [^18^F]Darapladib, a Lp-PLA2 Ligand, as Potential PET Imaging Tool of Atherosclerosis. Acs. Med. Chem. Lett..

[39] Zarganes-Tzitzikas T., Clemente G.S., Elsinga P.H., Dömling A. (2019). MCR Scaffolds Get Hotter with ^18^F-Labeling. Molecules.

[40] Mohammad S., Nguyen H., Nguyen M., Abdel-Rasoul M., Nguyen V., Nguyen C.D., Nguyen K.T., Li L., Kitzmiller J.P. (2019). Pleiotropic Effects of Statins: Untapped Potential for Statin Pharmacotherapy. Curr. Vasc. Pharmacol..

[41] Fatehi Hassanabad A., McBride S.A. (2019). Statins as Potential Therapeutics for Lung Cancer: Molecular Mechanisms and Clinical Outcomes. Am. J. Clin. Oncol..

[42] Fier P.S., Luo J., Hartwig J.F. (2013). Copper-Mediated Fluorination of Arylboronate Esters. Identification of a Copper(III) Fluoride Complex. J. Am. Chem. Soc..

[43] Refat M.S., Al-Saif F.A. (2015). Synthesis, spectral, thermal, and antimicrobial studies of transition metal complexes of atorvastatin calcium as a lipid-lowering agent. J. Therm. Anal. Calorim..

[44] Hatcher L.Q., Karlin K.D. (2006). Ligand Influences in Copper-Dioxygen Complex-Formation and Substrate Oxidations. Advances in Inorganic Chemistry.

[45] Novozhilov Y.V., Dorogov M.V., Blumina M.V., Smirnov A.V., Krasavin M. (2015). An improved kilogram-scale preparation of atorvastatin calcium. Chem. Cent. J..

[46] Bowden G.D., Pichler B.J., Maurer A. (2019). A Design of Experiments (DoE) Approach Accelerates the Optimization of Copper-Mediated ^18^F-Fluorination Reactions of Arylstannanes. Sci. Rep..

