# Supplementary material for: Late-Stage Copper-Catalyzed Radiofluorination of an Arylboronic Ester Derivative of Atorvastatin
Source: Molecules. 2019 Nov 20;24(23):4210. doi: 10.3390/molecules24234210 (PMC6930542; doi:10.3390/molecules24234210)
Supplement: Supplementary file 1 [file molecules-24-04210-s001.pdf]

SUPPORTING INFORMATION

Late-stage copper-catalyzed radiofluorination of an arylboronic ester derivative of atorvastatin

Gonçalo S. Clemente [1,†], Tryfon Zarganes-Tzitzikas [2,†], Alexander Dömling [2] and Philip H. Elsinga [1,*]

[1]Department of Nuclear Medicine and Molecular Imaging, University Medical Center Groningen, University of Groningen, The Netherlands
[2]Department of Drug Design, Groningen Research Institute of Pharmacy, University of Groningen, The Netherlands
*Correspondence: p.h.elsinga@umcg.nl; Tel.: +31-50-3613247
[†]These authors contributed equally.

Table of contents

Figure S1. ^{1}H NMR characterization of the Bpin labeling precursor 2

Figure S2. ^{13}C NMR characterization of the Bpin labeling precursor 3

Figure S3. Chromatographic profile of the compounds used and synthesized 4

Figure S4. Chromatographic profile of [^{18}F]atorvastatin spiked with reference 5

Table S1: Influence of azeotropic drying procedure in [^{18}F]F^- availability to the radiolabeling reaction 5

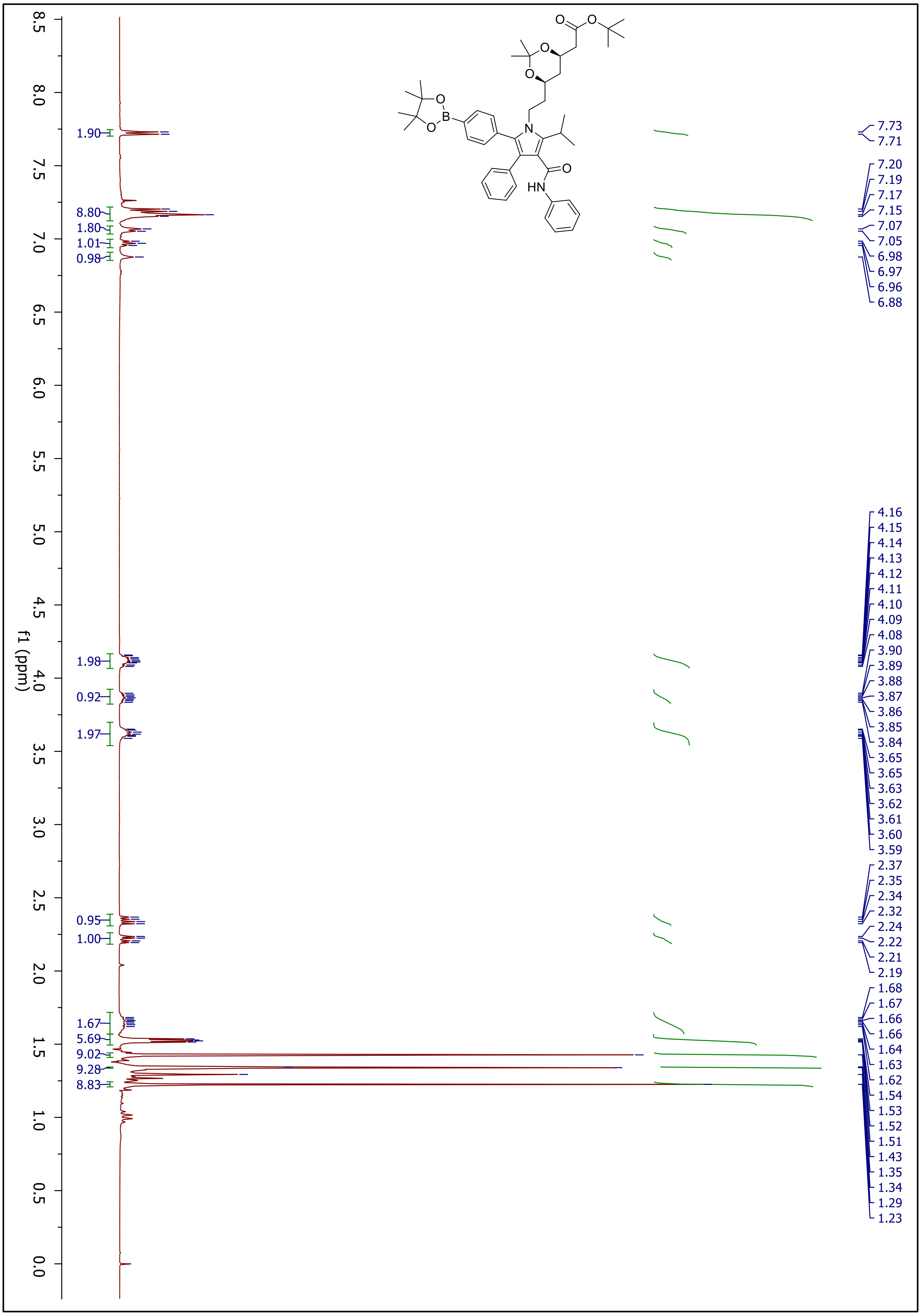

Figure S1. ^{1}H NMR characterization of the Bpin labeling precursor (**6**).

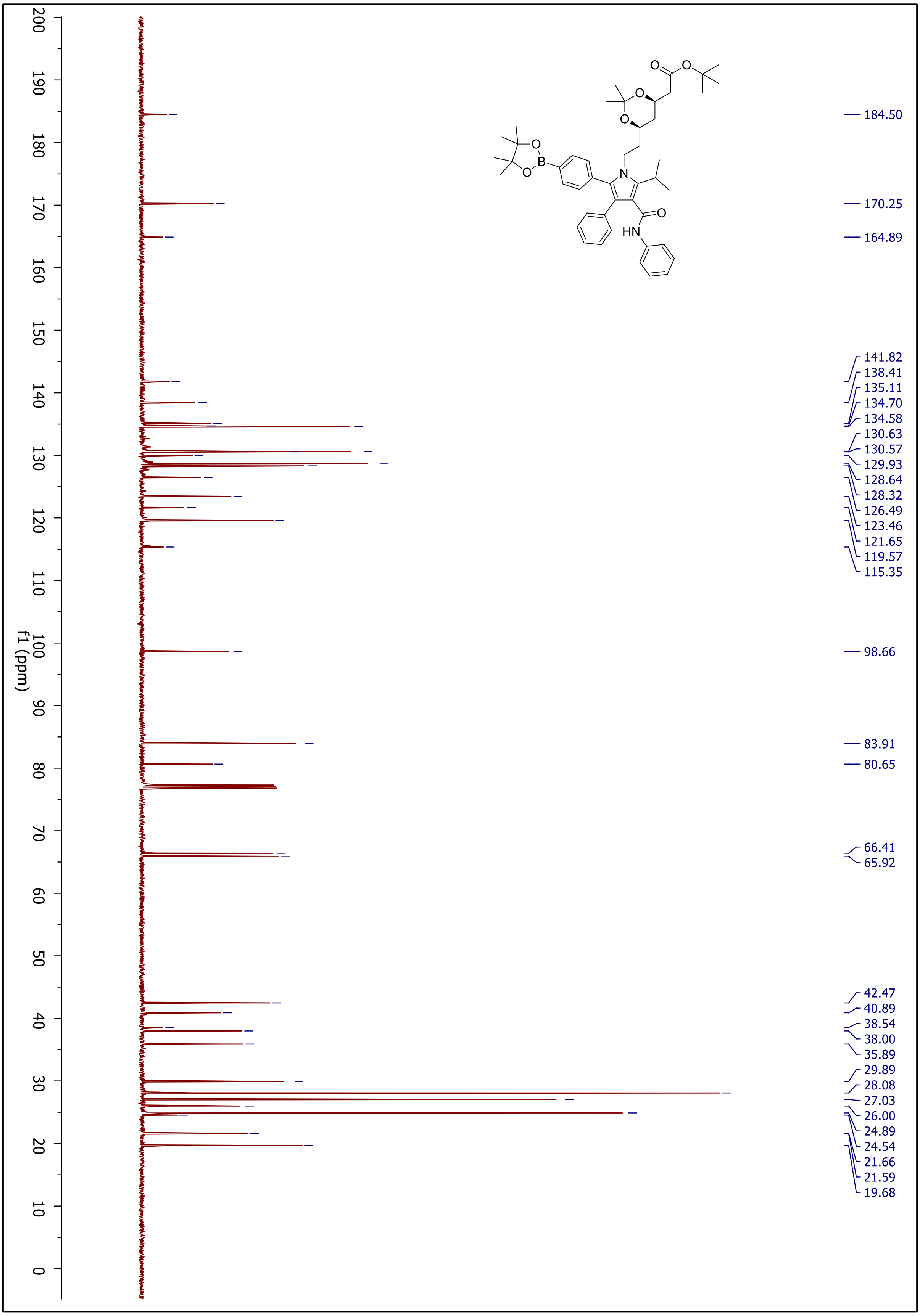

Figure S2. ^{13}C NMR characterization of the Bpin labeling precursor (**6**).

A)

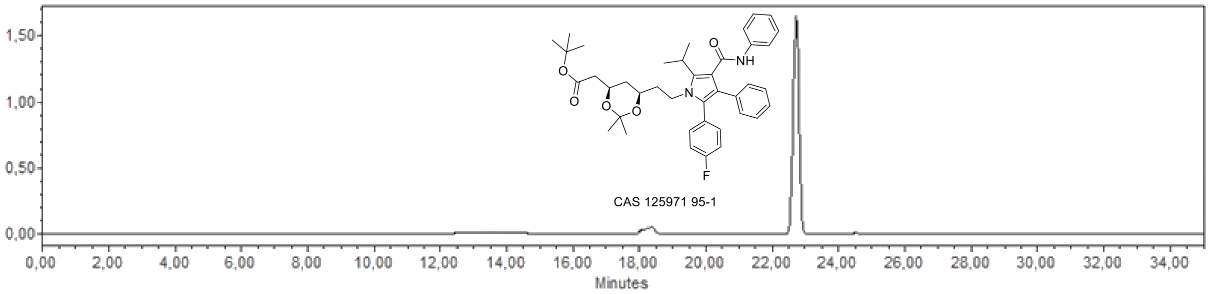

B)

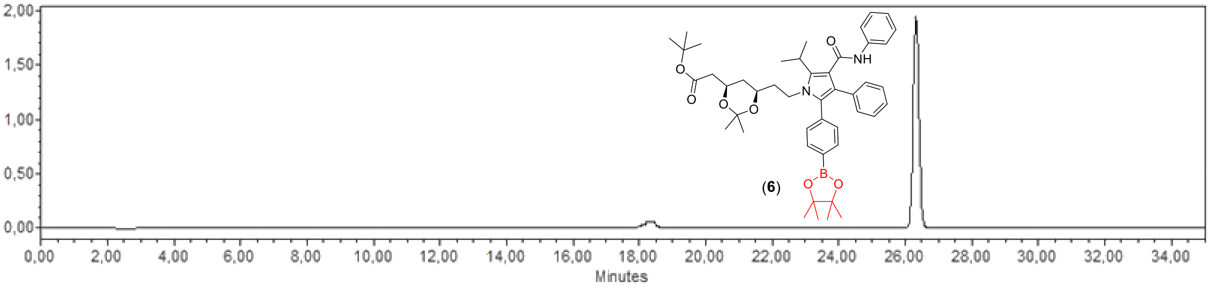

C)

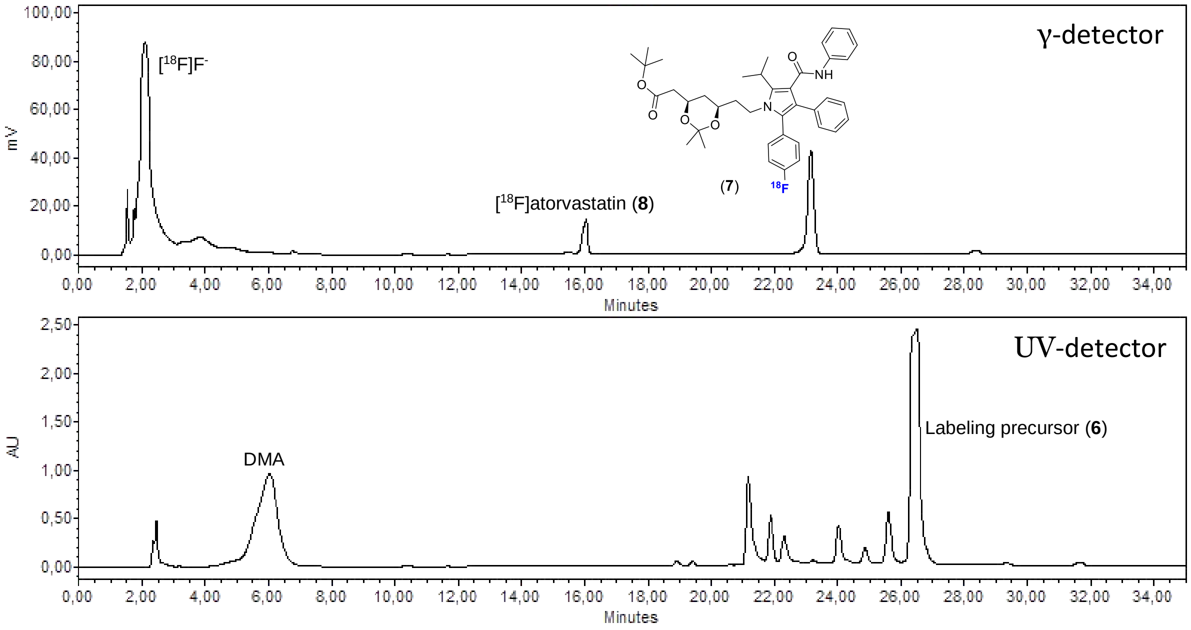

D)

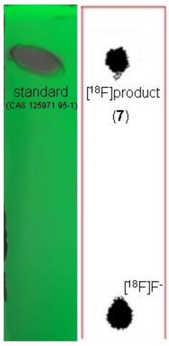

Figure S3. Chromatographic profile of the compounds. **A)** HPLC of atorvastatin intermediate standard; **B)** HPLC of Bpin labeling precursor (**6**); **C)** radio-HPLC of radiofluorinated compound; **D)** TLC and radio-TLC profile of the standard atorvastatin intermediate and radioactive species.

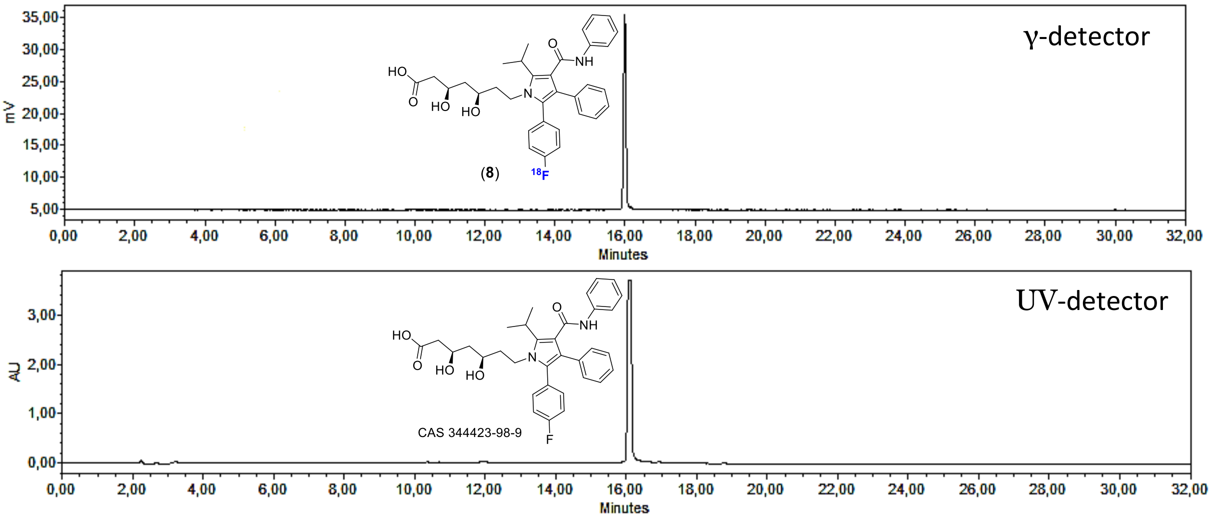

Figure S4. Chromatographic profile of [^{18}F]atorvastatin (**8**) spiked with reference (CAS 344423-98-9).

Table S1. Influence of azeotropic drying procedure in [^{18}F]F$^-$ availability to the radiolabeling reaction.

Azeotropic drying procedure	% [^{18}F]F$^-$ losses	
	% activity evaporated[c]	% adsorbed to vial[d]
105 °C, avoiding complete drying of [(Krypt-2.2.2)K$^+$][^{18}F]F$^-$ solution[a]	2.4% + 1.7%	17.7% + 2.6%
125 °C, avoiding complete drying of [(Krypt-2.2.2)K$^+$][^{18}F]F$^-$ solution[a]	12.4% + 2.3%	28.8% + 4.9%
105 °C, with [(Krypt-2.2.2)K$^+$][^{18}F]F$^-$ solution being fully dried[b]	3.2% + 0.9%	46.1% + 10.1%

[a][(Krypt-2.2.2)K$^+$][^{18}F]F$^-$ solution was azeotropically dried at 105/125 °C under gentle stirring and a light stream of argon without ever letting the mixture to completely dry (3 drying cycles with 0.5 mL anhydrous acetonitrile, each one starting after the previous volume has almost but not completely vanished, followed by dilution with 800 µl of anhydrous DMA immediately after the last cycle has nearly evaporated completely) (*n*=3); [b][(Krypt-2.2.2)K$^+$][^{18}F]F$^-$ solution was azeotropically dried at 105 °C under gentle stirring and a light stream of argon (3 drying cycles with 0.5 mL anhydrous acetonitrile, each one starting after the previous volume has completely vanished, followed by dilution with 800 µl of anhydrous DMA after the last cycle has evaporated completely) (*n*=3); [c]calculated by measuring the activity evaporated and trapped in 2 consecutive Sep-Pak Alumina N Plus Long cartridges placed in a ventilation needle at the vial; [d]calculated by measuring the activity remaining in the vial after emptying and washing 3 times with 0.8 mL DMA.
